# Biological Behavior of Bioactive Glasses SinGlass (45S5) and SinGlass High (F18) in the Repair of Critical Bone Defects

**DOI:** 10.3390/biom15010112

**Published:** 2025-01-13

**Authors:** Dayane Maria Braz Nogueira, Marcelie Priscila de Oliveira Rosso, Paulo Sérgio da Silva Santos, Manoel Damião Sousa-Neto, Alice Corrêa Silva-Sousa, Cleverson Teixeira Soares, Carlos Henrique Bertoni Reis, Jéssica de Oliveira Rossi, Cleuber Rodrigo de Souza Bueno, Daniela Vieira Buchaim, Rogério Leone Buchaim, Mariana Schutzer Ragghianti Zangrando

**Affiliations:** 1 Department of Anatomy, Faculty of Higher Education of the Interior of São Paulo (FAIP), Marília 17512-130, Brazil; dayanenogueira@usp.br; 2 Department of Physiotherapy, Physical Education, and Biomedicine, Estácio de Sá School of Ourinhos (FAESO), Ourinhos 19907-510, Brazil; marcelierosso@alumni.usp.br; 3 Department of Surgery, Stomatology, Pathology, and Radiology, Bauru School of Dentistry, University of São Paulo, Bauru 17012-901, Brazil; paulosss@fob.usp.br; 4 Department of Restorative Dentistry, School of Dentistry of Ribeirão Preto, University of São Paulo, Ribeirão Preto 14040-904, Brazil; sousanet@forp.usp.br (M.D.S.-N.); alicesousa@usp.br (A.C.S.-S.); 5 Lauro de Souza Lima Institute (ILSL), Anatomical Pathology Laboratory of Bauru (ANATOMED), Bauru 17034-971, Brazil; clev.blv@terra.com.br; 6 Beneficent Hospital (HBU), University of Marilia (UNIMAR), Marilia 17525-160, Brazil; dr.carloshenriquereis@usp.br; 7 Occupational Medicine, Marilia School of Medicine (FAMEMA), Marilia 17519-030, Brazil; jessica.or@usp.br; 8 Anatomy Department, Faculty of Dentistry, University Center of Adamantina (FAI), Adamantina 17800-000, Brazil; cleuberbueno@fai.com.br; 9 Anatomy Department, Medical School, University Center of Adamantina (FAI), Adamantina 17800-000, Brazil; danibuchaim@alumni.usp.br; 10 Graduate Program in Anatomy of Domestic and Wild Animals, Faculty of Veterinary Medicine and Animal Science, University of São Paulo (FMVZ/USP), Sao Paulo 05508-270, Brazil; 11 Department of Biological Sciences, Bauru School of Dentistry (FOB/USP), University of São Paulo, Bauru 17012-901, Brazil; 12 Department of Prosthodontics and Periodontics, Bauru School of Dentistry, University of São Paulo, Bauru 17012-901, Brazil; mariana@fob.usp.br

**Keywords:** bone regeneration, bioactive glass, SinGlass, SinGlass High (F18), osteogenesis, biomaterials, critical defects

## Abstract

This study evaluated the osteogenic potential of the bioactive glasses SinGlass (45S5) and SinGlass High (F18) in regenerating critical bone defects in rat calvaria. Both biomaterials promoted new bone formation around the particles, with the SinGlass High (F18) group exhibiting a higher rate of bone maturation. Histomorphological and birefringence analyses revealed better organization of the newly formed bone in the biomaterial-treated groups, and immunohistochemistry indicated the expression of osteogenic markers such as osteocalcin, immunostaining for bone morphogenetic protein 2 (BMP 2), and immunostaining for bone morphogenetic protein 4 (BMP 4). Microtomography computadorized (Micro-CT) revealed centripetal bone formation in both groups, with greater integration of the particles into the surrounding bone tissue. The superior performance of SinGlass High (F18) was attributed to its higher potassium and magnesium content, which enhance osteoconductivity. After 42 days, the SinGlass High (F18) group showed the highest percentage of new bone formation, in line with previous studies. Although our results are promising, the limited follow-up period and use of a single animal model highlight the need for further research to validate clinical applicability. SinGlass High (F18) appears to be a viable alternative to autografts in bone repair, with potential to improve tissue integration and accelerate recovery.

## 1. Introduction

Biomaterials are attracting increasing interest from researchers across various fields. In dentistry, for example, biomaterials have been extensively studied in hard (e.g., bones) [1,2,3,4,5,6,7,8] and soft tissues [9,10]. Bioactive materials can activate immune cells and body proteins, thereby offering great potential for regenerating durable tissues. Among these materials, bioactive glass (BG) [11,12,13] is particularly notable for its unique properties, which enable many clinical applications in tissue regeneration in both dentistry and medicine [14,15,16,17].

In dentistry, BGs are used for pulp capping, root canal treatment, restorations, mineralizing agents, implant coatings, 3D scaffolds, and air abrasion [12,13,18,19,20]. Moreover, they exhibit remineralizing effects and antimicrobial properties, aiding in the management of oral diseases such as periodontitis and caries [17,21]. In medicine, their applications extend from orthopedics to soft tissue restoration [22,23,24].

Bone regenerative therapy aims to restore tissue morphology and function using osteoinductive growth factors, osteogenic cells, osteoconductive structures, bioactive molecules, or combinations with biomaterials [25,26,27]. The clinical use of polymeric scaffolds, with or without cellular or biological mediators, is well documented in regenerative therapies [28,29].

In 1992, PerioGlas^®^ (NovaBone, Alachua, FL, USA) became the first particulate to be BG approved by the Food and Drug Administration for reconstructing mandibular and periodontal defects [30,31]. Initially used to aid bone regeneration around tooth extraction sites, it also improved bone quality for titanium implants. Its clinical success culminated in its official introduction in 1999 as a particulate bone graft material for non-load-bearing orthopedic applications. Comparative studies have subsequently shown that PerioGlas^®^ exhibits lower risks of infection and mechanical failure than autografts [32], while eliminating the need for a local donor site [33].

In addition to osteogenesis, products derived from the degradation of BG promote chondrogenesis [34,35]. Other dental and maxillofacial applications include bone growth stimulation [23,36], pulp capping [37], orbital floor fracture repair [34], frontal sinus obliteration [38], and alveolar bone regeneration [12]. In vivo studies suggest that bioglass 45S5 may also serve as an injectable treatment for urinary incontinence [39].

Although some biopolymers support cell viability, many cannot self-replicate, differentiate, or migrate, which can lead to early cell death. Therefore, BGs must be passively and actively biocompatible. The BGs are biomaterials that support cell survival and promote critical interactions, such as attachment, migration, differentiation, and proliferation for effective biological functioning [7,28,40,41,42,43].

The industry is developing various alloplastic materials that offer reduced costs and enhanced clinical applicability in regenerative medicine. Thus, we carried out an unprecedented comparative biological test of two bioactive glasses, one of which is commercially available (45S5) and the other in the experimental phase (F18).

Therefore, this study aimed to evaluate and compare the efficacy of two biomaterials, SinGlass 45S5 and SinGlass High (F18), in the repair of critical bone defects in rat calvaria.

## 2. Materials and Methods

### 2.1. Bioglass

SinGlass 45S5 is a biomaterial made from calcium silicate, sodium, and amorphous phosphorus, known as the 45S5 formulation. Its composition includes 45% SiO_2_, 24.5% Na_2_O, 24.5% CaO, and 6% P_2_O_5_ (wt%), as per ASTM F1538-03 (2017), which specifies the physicochemical and biocompatibility requirements for BGs used in implants. SinGlass High (F18) has the same indications as SinGlass 45S5 but differs in composition. It is made from calcium silicate, sodium, and amorphous phosphorus, collectively referred to as the F18 formulation. This biomaterial belongs to the SiO_2_–Na_2_O–CaO–P_2_O_5_–K_2_O–MgO system and is protected under patent BR10 INPI 20130209619. Details on its physicochemical characterization are described elsewhere [44,45]. We used BGs with a particle size of 0.355–0.500 µm.

SinGlass 45S5 and SinGlass High (F18) were supplied by Síntegra Surgical Sciences Ltd.a. (Pompeia, São Paulo, Brazil) in collaboration with Vetra Biomaterials Ltd.a. (Ribeirão Preto, São Paulo, Brazil). Both were produced using the conventional melting method. Briefly, raw materials were mixed and homogenized in a platinum crucible and then fused in a bottom-loading furnace (Nabertherm GmbH, Lilienthal, Germany) at 1450 °C. The particles were ground and sieved to achieve a particle size of 355–500 µm and were sterilized using gamma radiation.

In the description of the materials and methods and results, when describing biomaterial, we are referring to SinGlas 45S5 and SinGlass High (F18). When necessary, we differentiated each one.

### 2.2. Experimental Design

This study followed the ethical principles outlined by the Brazilian College of Animal Experimentation and was approved by the Animal Ethics Committee (process no. 001/2022) of the Bauru School of Dentistry, University of São Paulo. This study adhered to ARRIVE (Animal Research: Reporting of In Vivo Experiments) [46] and NC3Rs (National Centre for the Replacement, Refinement, and Reduction of Animals in Research) guidelines to ensure ethical standards for animal experimentation.

Rats were closely monitored for signs of pain or behavioral changes, such as apathy, depression, aggression, or hyperactivity. Changes in locomotion, posture, facial expressions, feeding, and water consumption were also monitored, with close attention to changes in clinical signs.

We used 42 male Wistar rats (*Rattus norvegicus*), aged 90 days and averaging 300 g. They were housed in conventional cages (four per cage) with unrestricted access to water and food, under a 12 h light cycle at an average temperature of 21 °C, monitored using a thermometer.

**Figure 1:**
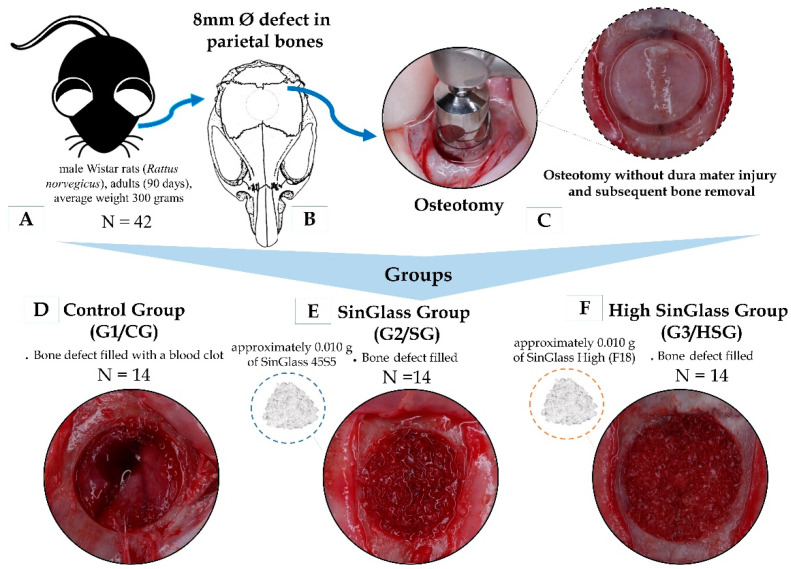
Schematic of the experimental design. (**A**)—number of animals used in the research and their characteristics; (**B**)—model of the location of the bone defect performed in parietal bones; (**C**)—an 8 mm circular bone defect was created using a trephine drill attached to a contra-angle handpiece connected to a low-speed electric motor (20,000 rpm). Separation of animals into groups: (**D**)—Control Group (G1/CG): bone defect filled with a blood clot; (**E**)—SinGlass Group (G2/SG): bone defect filled with approximately 0.010 g of SinGlass 45S5; (**F**)—High SinGlass Group (G3/HSG): bone defect filled with approximately 0.010 g of SinGlass High (F18).

Rats were randomly assigned to three groups (*n* = 14 per group; Figure 1): Control Group (G1/CG) with calvarial bone defects filled with a local blood clot; SinGlass Group (G2/SG) with defects filled with approximately 0.010 g of SinGlass 45S5; and High SinGlass Group (G3/HSG) with defects filled with approximately 0.010 g of SinGlass High (F18).

### 2.3. Surgical Procedures

The rats were anesthetized intraperitoneally in the right lower quadrant with ketamine hydrochloride (80 mg/kg; Dopalen^®^, Sespo Indústria e Comércio Ltd.a., São Paulo, Brazil) and xylazine hydrochloride (10 mg/kg; Anasedan^®^, Sespo Indústria e Comércio Ltd.a., São Paulo, Brazil). The anesthetic was administered under strict monitoring.

The parietal region was shaved and then antiseptically treated with a topical solution of PovidoneIodine (Povidine^®^ Antiseptic, Vic Pharma Ind and Comércio Ltd., São Paulo, Brazil) at 10%. Thirty minutes before surgery, the rats received subcutaneous injections of tramadol hydrochloride (50 mg; Tramal^®^, Pfizer, Itapevi, Brazil) at 17.8 mg/kg and meloxicam (15 mg; EMS, Hortolândia, Brazil) at 1.5 mg/kg. The rats were secured in a ventral position on the operating table, and local anesthesia was applied with 2% lidocaine (Xylestesin^®^, 20 mL, injectable solution, Cristália Produtos Químicos Farmacêuticos Ltd.a., São Paulo, Brazil) at 0.2 mL at the incision site to control pain and maintain homeostasis.

A 4 cm semilunar incision was made in the skin overlying the cranial vault, and the periosteum and adjacent tissues were separated to expose the parietal bones. An 8 mm circular bone defect was created using a trephine drill (Neodent^®^, Curitiba, Brazil) attached to a contra-angle handpiece (NSK, Suzano, Brazil) connected to a low-speed electric motor (20,000 rpm). Continuous irrigation with sterile 0.9% saline was performed throughout the procedure to prevent bone necrosis and protect the dura mater and brain. After the defect was created, the tissues were repositioned and sutured with 4-0 silk thread (Ethicon, Johnson & Johnson Company, São Paulo, Brazil). All procedures were conducted by a single operator to ensure consistent conditions for the animals.

After surgery, rats received subcutaneous injections of tramadol (50 mg; Tramal^®^, Farmaceutici Formenti S.p.A., Origgio, Italy) at 17.8 mg/kg and meloxicam (15 mg; Meloxicam^®^, Sanofi Medley, Campinas, Brazil) at 1.5 mg/kg every 12 h for three days. After this period, they were given 4 mL of dipyrone 500 mg/mL diluted in 396 mL of water, (Dipirona Analgex V^®^, Agener União, São Paulo, Brazil) in their drinking water for five days.

Seven rats from each experimental group were euthanized 14 and 42 days post-surgery, and the calvariae were collected immediately.

### 2.4. Micro-CT Scanning

The rat calvariae were scanned using a high-resolution scanner (Skyscan 1174 micro-CT SkyScan, Kontich, Belgium) at the Research Laboratory of Endodontics, Department of Restorative Dentistry, School of Dentistry of Ribeirão Preto, University of São Paulo. Each specimen was secured with wax in a metal holder and mounted on a rotating stage inside the micro-CT scanner, oriented perpendicular to the radiation source. Scanning was conducted at 50 kV and 800 mA, achieving an isotropic resolution of 17.39 μm, with a 180° rotation around the vertical axis, a rotation step of 0.7°, and the acquisition of 2 frames. The X-ray beam was filtered using a 0.5 mm aluminum filter. Axial slices were reconstructed using NRecon v. 1.7.4.4 software (Bruker-microCT, Karlsruhe, Germany). The scanning parameters included: 17.39 μm resolution, a rotation step of 0.7°, 360° = off, random movement = off, frame = 2, and a 0.5 mm aluminum filter. After reconstruction, the images of each specimen were analyzed using CTAn v. 1.18 software (Bruker-microCT, Karlsruhe, Germany) for quantitative 2D and 3D analysis. Qualitative analysis of the newly formed bone tissue was conducted using CTVox software, v. 1.18 software (Bruker-microCT, Karlsruhe, Germany).

### 2.5. Histotechnical Processing

After acquiring micro-CT images, the samples were fixed in 10% buffered formalin for 72 h, washed in running water for 24 h, and then demineralized in 10% ethylenediaminetetraacetic acid (EDTA), with weekly solution changes. Demineralization was monitored using radiographs taken with a Kodak Insight Occlusal film (Kodak, Eastman Co., Rochester, NY, USA).

Histological processing followed a standardized protocol for embedding in Histosec^®^ (synthetic resin and paraffin) (Merck, Hessen, Germany) and for preparing longitudinal semi-serial sections at 50 µm intervals. Sections of 4 µm were used for immunohistochemistry, and 5 µm sections were stained with hematoxylin and eosin (HE) and Masson’s trichrome. All sections were obtained using a Leica RM2245 microtome (Leica, Wetzlar, Germany).

HE-stained slides were used for histomorphometry, and Picrosirius red-stained slides were used to evaluate collagen fibers. Additional sections were prepared for immunolabeling of vascular endothelial growth factor (VEGF), tartrate-resistant acid phosphatase (TRAP), osteocalcin (OCN), and bone morphogenetic proteins 2 and 4 (BMP 2 and BMP 4).

### 2.6. Immunohistochemical Processing

Histological sections were prepared on silanized slides based on the immunohistochemical map (Dako, São Bernardo do Campo, Brazil). Sections (3–4 µm thick) were fixed, deparaffinized, and rehydrated using a standardized protocol. Before oven processing, sections were dried and fixed at 70 °C for 60 min. Deparaffinization was conducted using an automated system (LEICA, Wetzlar, Germany), with slides sequentially immersed in two xylene baths (5 min each) and four absolute ethanol baths, followed by rehydration in running water for 5 min.

Antigen retrieval and slide loading were managed using DAKO software (v. TRD 1.3, Copenhagen, Denmark), which also oversaw retrieval solution replacement (valid for three cycles) and confirmed process completion. Marker separation followed the immunohistochemical map, including antibodies, hydrogen peroxide, polymer, DAB + Chromogen, EnVision Flex Mouse, and EnVision Flex Rabbit. The antibodies used were BMP-2 Ab (AF5163, lot 15v8605, Affinity Bioreagents, Golden, CO, USA), VEGFA Ab (AF5131, lot 63m6093, Affinity Bioreagents, Golden, CO, USA), BMP-4 Ab (AF5175, Affinity Bioreagents, Golden, CO, USA), OCN Ab (DF12303, lot 85n2245, Affinity Bioreagents, Golden, CO, USA), and ACP-5 Ab (DF6989, lot 70b0275, Affinity Bioreagents, Golden, CO, USA).

### 2.7. Histological Evaluation

The calvarial defect was thoroughly assessed for granulation tissue, inflammatory infiltrate, the quality of newly formed tissue (immature or mature/lamellar bone), and the degree of defect filling. Five semi-serial sections of the surgical bed from each defect were analyzed using an Olympus BX50 light microscope (Olympus Corporation, Tokyo, Japan). Images were captured at 4× and 40× magnifications using an Olympus DP 71 digital camera (Olympus Corporation, Tokyo, Japan) attached to the microscope and quantitatively analyzed using ImageJ^®^ software version 1.50d (Wayne Rasband, National Institutes of Health, Bethesda, ML, USA).

### 2.8. Birefringence Analysis of Collagen Content

To assess the birefringence intensity of collagen fibers, histological sections were stained with picrosirius red and counterstained with Harris hematoxylin. Observations were made using a polarized lens attached to a Leica^®^ DM IRB/E inverted microscope at the Integrated Research Center (Bauru School of Dentistry, University of São Paulo). Full images of the bone defect were captured at 10× magnification, enabling the identification of collagen fibers undergoing repair, ranging from the thinnest fibers (exhibiting birefringence in yellow tones) to the thickest ones (in red tones). To standardize the analyses, light intensity and the angle of the polarized lens (90° to the light source) were kept constant across all images. Birefringence intensity was quantified using KS 300/400 AxioVision software (version 4.8, Carl Zeiss, Jena, Germany), and pixel^2^ values were averaged and analyzed statistically.

### 2.9. Statistical Analysis

The histomorphometric data were assessed for normality using the Kolmogorov–Smirnov test and for homoscedasticity using the Bartlett test. The percentages of newly formed bone and reactive tissue were compared among groups using analysis of variance (ANOVA) followed by Tukey’s test for multiple comparisons. For the percentage of biomaterial, an unpaired Student’s *t*-test was performed. All analyses were conducted using GraphPad Prism software version 8.0 (GraphPad^®^ Software, La Jolla, CA, USA), with statistical significance set at *p* < 0.05. Values are reported as mean ± standard deviation.

## 3. Results

The experiment was conducted as planned, strictly following the protocol for animal medication and monitoring. By day 14, we observed skin tissue recovery, wound healing, and fur regrowth.

### 3.1. Micro-CT Analysis

Micro-CT images were qualitatively analyzed, and quantification was performed by assessing histological slides due to the radiopacity similarities between new bone and SinGlass 45S5 and SinGlass High (F18) (biomaterial) particles.

**Figure 2:**
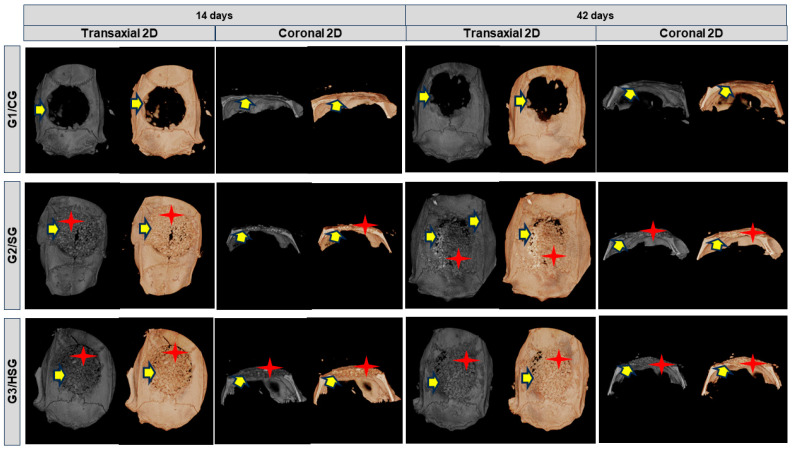
Reconstructed two-dimensional (2D) micro-CT images (transverse and coronal sections) of rat calvarial bone defects with 8 mm at 14 and 42 days. Control Group (G1/CG): bone defect filled with a blood clot. SinGlass Group (G2/SG): bone defect filled with approximately 0.010 g of SinGlass 45S5. High SinGlass Group (G3/HSG): bone defect filled with approximately 0.010 g of SinGlass High (F18). Yellow arrows indicate newly formed bone, and red stars indicate the biomaterial particles.

By day 14 (Figure 2), all three experimental groups exhibited a centripetal pattern of bone formation. This pattern was marked by a gradual increase in bone tissue density, visible as grayscale gradients in the peripheral areas of the bone defect. Biomaterial particles were surrounded by immature bone tissue.

By day 42, all groups showed increased immature bone, but defect closure was incomplete, confined to the edges of the residual bone, with some mineralized tissue. Bone remodeling mainly occurred at the periphery of the defect (based on variations in tissue density), whereas the central area remained filled with biomaterial particles.

### 3.2. Histomorphological Analysis

**Figure 3:**
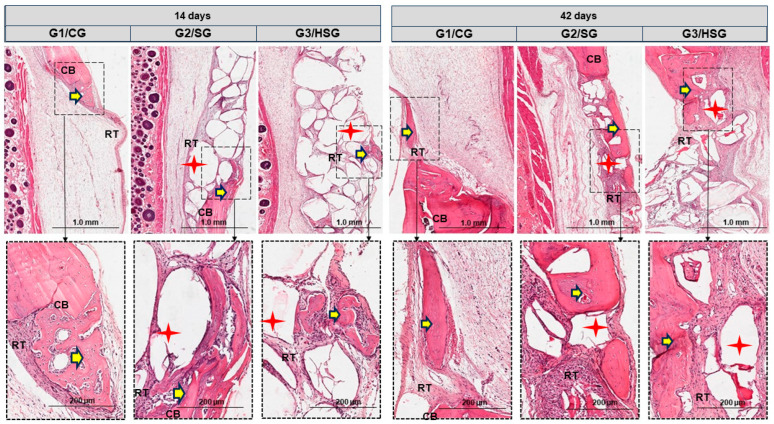
Longitudinal histological sections (hematoxylin-eosin) of calvarial bone defects at 14 and 42 days. Control Group (G1/CG): bone defect filled with a blood clot. SinGlass Group (G2/SG): bone defect filled with approximately 0.010 g of SinGlass 45S5. High SinGlass Group (G3/HSG): bone defect filled with approximately 0.010 g of SinGlass High (F18). The cortical defect is located on the lateral side of all images. Cortical bone (CB), reaction tissue (RT), biomaterial particles (red stars), and new bone (yellow arrows). Original magnification: 4×, scale bar = 1 mm. Detail: 20×, scale bar = 200 µm.

**Figure 4:**
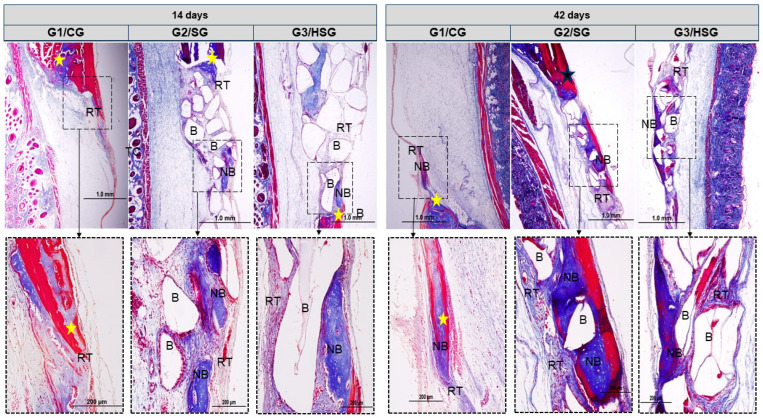
Longitudinal histological sections (Masson’s trichrome) of calvarial bone defects at 14 and 42 days. Control Group (G1/CG): bone defect filled with a blood clot. SinGlass Group (G2/SG): bone defect filled with approximately 0.010 g of SinGlass 45S5. High SinGlass Group (G3/HSG): bone defect filled with approximately 0.010 g of SinGlass High (F18). Note the deposition of osteoid matrix at the edges of the defect (yellow stars), biomaterial particles (B) interspersed with densely cellular reaction tissue (RT), and new bone (NB). The calvarial defect is located on the lateral side of all images. Original magnification: 4× scale bar = 1 mm. Detail: 20×, scale bar = 200 µm.

At 14 and 42 days, all groups showed cortical bone remodeling, but only the G2/SG and G3/HSG biomaterial particles. All groups showed reaction tissue and isolated areas of new bone formation. The G2/SG and G3/HSG exhibited a transition from loose to dense reaction tissue around the biomaterial particles, along with immature bone formation (Figure 3 and Figure 4).

By day 42, the G2/SG and G3/HSG showed surface irregularities in the biomaterials, attributed to absorption, along with a considerable increase in new bone formation surrounding the particles. The G3/HSG showed a larger area of new bone formation, with physical characteristics suggesting greater bone maturation. All groups exhibited dense reaction tissue and advanced cortical bone remodeling, particularly in the G3/HSG group (Figure 3 and Figure 4).

### 3.3. Birefringence of Collagen Fibers

**Figure 5:**
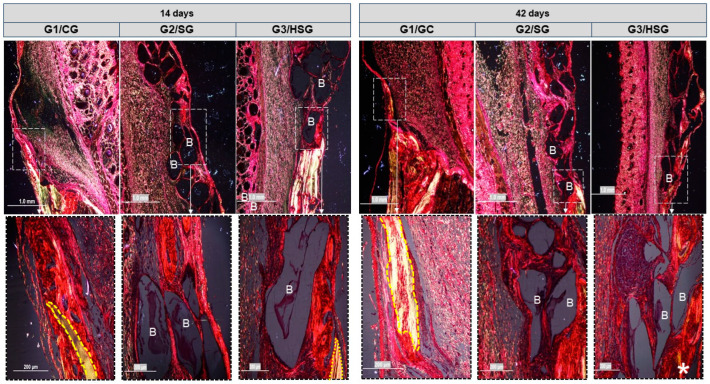
Longitudinal histological sections (Picrosirius red under polarized light) of calvarial bone defects at 14 and 42 days. Control Group (G1/CG): bone defect filled with a blood clot. SinGlass Group (G2/SG): bone defect filled with approximately 0.010 g of SinGlass 45S5. High SinGlass Group (G3/HSG): bone defect filled with approximately 0.010 g of SinGlass High (F18). RGB spectrum (green-yellow-red) analysis identified collagen fibers: mature bone and type I collagen (yellow-green); immature bone and type III collagen (reddish). The yellow dashed line delimits the defect edge, and asterisks indicate collagen fibers at advanced maturation stages. Biomaterial particles (B) (dark background). Original magnification: 4×, scale bar = 1 mm (Images in the top row). Detail: 20×, scale bar = 200 µm (Images in the bottom row).

At 14 days, all groups exhibited thin, disorganized collagen fibril bundles characteristic of type III collagen. These fibrils formed linear trabeculae across the wound and around the biomaterial particles. The birefringence pattern in the recipient bed varied from reddish-orange to greenish. The G2/SG showed areas of recent mineralization, with collagen fibers transitioning to yellow-green birefringence centrally and juxtaposed with the biomaterial particles (Figure 5).

At 42 days, collagen fibers thickened into lamellar bundles characteristic of type I collagen. These fibers were oriented parallel and circumferentially around the biomaterial particles, with birefringence ranging from green to yellow. In the G2/SG and G3/HSG, greenish birefringence predominated at the defect center, and the indistinct boundary between new and original bone indicated advanced bone maturation.

Picrosirius-red staining allowed the identification of collagen types and fiber arrangement in the reaction tissue. At 14 days, all groups exhibited disorganized type III collagen fibers, but the G3/HSG showed more advanced maturation with some mineralization. By 42 days, the collagen fibers were thicker and displayed a lamellar organization characteristic of type I collagen.

### 3.4. Immunohistochemical Analysis

**Figure 6:**
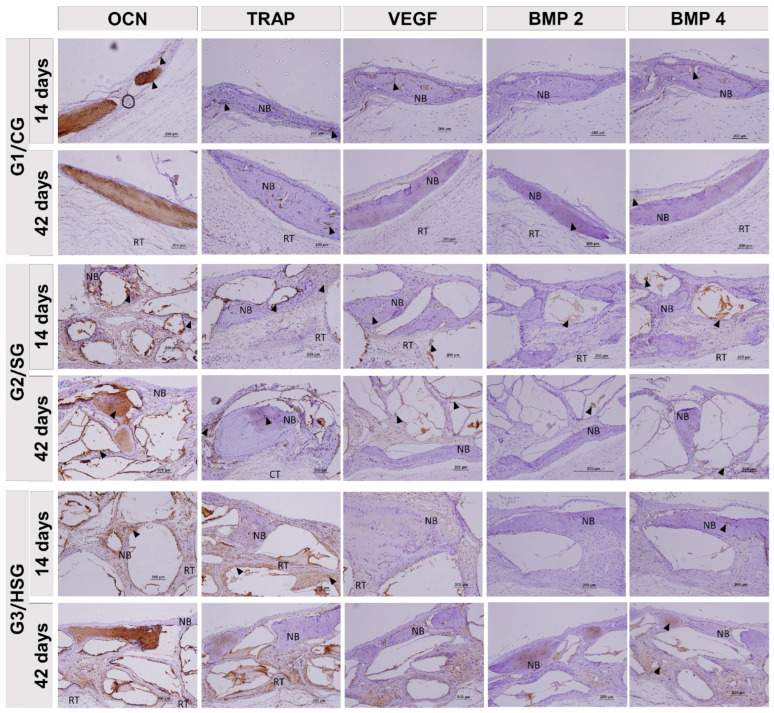
Histological sections (counterstained with Harris hematoxylin) showing immunolabeling in the three experimental groups at 14 and 42 days. Control Group (G1/CG): bone defect filled with a blood clot. SinGlass Group (G2/SG): bone defect filled with approximately 0.010 g of SinGlass 45S5. High SinGlass Group (G3/HSG): bone defect filled with approximately 0.010 g of SinGlass High (F18). Arrowheads indicate sites where brown stain marks the proteins: immunostaining for osteocalcin (OCN), immunostaining for tartrate-resistant acid phosphatase (TRAP), immunostaining for vascular endothelial growth factor (VEGF), immunostaining for bone morphogenetic protein 2 and 4 (BMP 2 and BMP 4). Positive cells’ reaction tissue (RT) and new bone (NB). Scale bars: 200 μm, magnification: 20×.

The immunohistochemical analysis identified immunomarkers (Figure 6), such as OCN and TRAP. Immunoreactive cells showed dark brown staining in various cellular structures, with TRAP staining confined to the cytoplasm and OCN, VEGF, BMP-2, and BMP-4 present in the cytoplasm and, to a lesser extent, in the extracellular matrix.

BMP 2 and BMP 4 showed more pronounced expression in the G2/SG and G3/HSG at 14 and 42 days, localized around the biomaterial particles, in the newly formed bone matrix and in the reaction tissue. In the G3/HSG, staining patterns were particularly evident at 42 days.

VEGF, an angiogenic immunomarker, exhibited more intense immunostaining at 42 days in the G2/SG and G3/HSG. OCN was consistently detected in the newly formed bone and around the biomaterial particles across all groups and time points.

### 3.5. Morphometric Analysis

**Figure 7:**
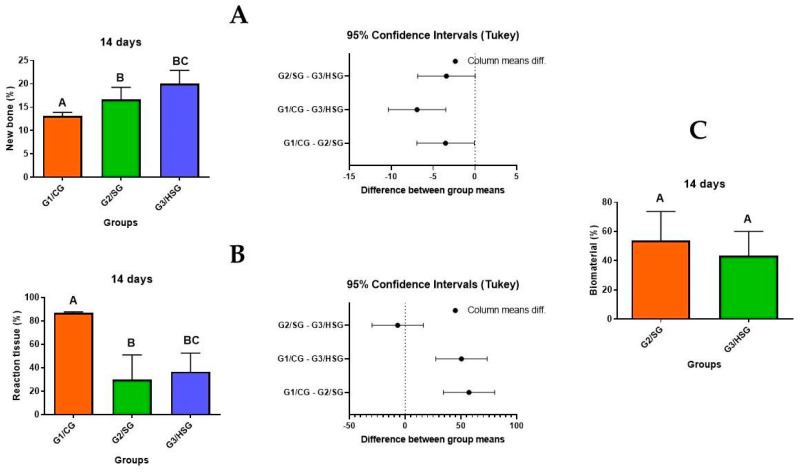
Percentages of (**A**) new bone, (**B**) reaction tissue, and (**C**) biomaterial particles at 14 days. Control Group (G1/CG): bone defect filled with a blood clot. SinGlass Group (G2/SG): bone defect filled with approximately 0.010 g of SinGlass 45S5. High SinGlass Group (G3/HSG): bone defect filled with approximately 0.010 g of SinGlass High (F18). Different letters indicate statistically significant differences among groups.

At 14 days, the percentage of new bone formation was similar between the G3/HSG and G2/SG, but significantly higher than the G1/CG (Figure 7A and Table 1). The amount of reaction tissue was significantly higher in the G1/CG than the G2/SG and G3/HSG, but similar between the G2/SG and G3/HSG (Figure 7B and Table 2). Lastly, the percentage of residual biomaterial was similar between the G2/SG and G3/HSG (Figure 7C and Table 3).

**Figure 8:**
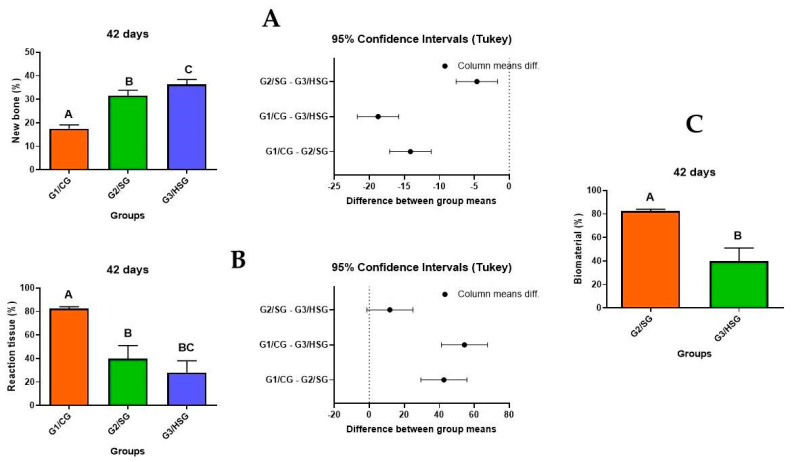
Percentages of (**A**) new bone, (**B**) reaction tissue, and (**C**) biomaterial particles at 42 days. Control Group (G1/CG): bone defect filled with a blood clot. SinGlass Group (G2/SG): bone defect filled with approximately 0.010 g of SinGlass 45S5. High SinGlass Group (G3/HSG): bone defect filled with approximately 0.010 g of SinGlass High (F18). Different letters indicate statistically significant differences among groups.

At 42 days, the percentage of new bone formation differed significantly across all groups, with the G3/HSG showing the highest percentage of new bone formation and the G1/CG the lowest percentage (Figure 8A and Table 1). The percentage of reaction tissue at 42 days followed the same pattern observed at 14 days, with significantly higher values in the G1/CG than the G2/SG and G3/HSG, but similar values between the G2/SG and G3/HSG (Figure 8B and Table 2). The percentage of residual biomaterial was significantly lower in the G3/HSG than the G2/SG (Figure 8C and Table 3), thus showing greater resorption of biomaterial particles in the G3/HSG.

## 4. Discussion

We evaluated and compared the performance of SinGlass 45S5 and SinGlass High (F18) in the regeneration of cranial bone defects in rats. Both biomaterials promoted bone formation around the particles; however, the group treated with SinGlass High (F18) (G3/HSG) showed a faster rate of bone maturation than the group treated with SinGlass 45S5 (G2/SG). We also found that the biomaterial-treated groups showed more organized new bone formation and pronounced expression of osteogenic markers, with G3/HSG inducing a more effective regenerative response.

Previous characterizations of biomaterials structurally similar to F18 have identified promising mechanical and osteoconductive properties [45,47,48]. The porous structure of SinGlass High (F18) facilitates cell infiltration and creates a favorable environment for bone regeneration, which are essential aspects for biomaterial integration. Silva Júnior et al. [44] reported that biomaterials, such as BGs, when applied to segmental bone defects, induced a controlled inflammatory response and moderate vascularization. These conditions are critical for osteogenesis, osteoid matrix formation, bone remodeling, and long-term material resorption.

Micro-CT analyses conducted at 14 and 42 days revealed centripetal bone formation in the G2/SG and G3/HSG, indicating that the biomaterials particles had progressively integrated into the bone tissue. These findings agree with the existing literature, which reports the bioactivity of BGs in promoting bone mineralization in critical defects [34,49,50,51]. This bioactivity contributes to cell proliferation and the expression of osteogenic markers, as observed in the groups treated with SinGlass 45S5 and SinGlass High (F18), suggesting that the mechanisms of action of these biomaterials are similar. The formation of new bone surrounding the biomaterial particles observed in this study indicates an ongoing bone remodeling process, which is expected given the evaluation periods (14 and 42 days). In contrast to G1/CG, which exhibited limited bone formation, the biomaterial-treated groups showed a higher density of newly formed bone tissue in the peripheral regions of the defect.

Our histomorphological findings corroborate the micro-CT results, demonstrating increased new bone formation in the G2/SG and G3/HSG. The transition from a loose reaction tissue to a denser tissue around the biomaterial particles, particularly in the G3/HSG, suggests a positive interaction between the biomaterial and the tissue microenvironment. There was no evidence of foreign body reaction in the two bioglasses used in this study. This interaction seems to favor the formation of a robust and structurally stable tissue. The superior bone maturation observed with SinGlass High (F18) compared to SinGlass 45S5 can be attributed to its distinct composition, which includes higher potassium (K_2_O) and magnesium (MgO) content, known to positively influence osteoconduction [11,29,44] (Figure S1, Supplementary Materials). This effect was also highlighted by Silva Júnior et al. [44], who reported that SinGlass High (F18) induces the formation of membranes rich in osteoid matrix and collagen fibers, promoting cell differentiation and the formation of denser, more integrated bone over time [45,52,53].

The birefringence analysis of collagen fibers reinforced the findings on bone maturation. The transition from type III collagen fibers (characteristic of immature bone) to type I collagen (indicative of mature bone), observed at 42 days, indicates advanced bone repair in the G2/SG and G3/HSG. Notably, this transition was accompanied by greater organization and thickening of the fibers in the G3/HSG. This observation is consistent with previous studies highlighting the ability of biomaterials, such as BGs, to stimulate collagen deposition and the formation of a well-organized bone matrix [7,41,54,55].

Immunohistochemical analysis revealed the expression of osteogenic markers (e.g., OCN) and a positive angiogenic response, as evidenced by the more pronounced presence of VEGF at 42 days in the biomaterial-treated groups. These markers are important for effective tissue regeneration and stable, bone matrix formation [4,56]. OCN marks osteoblastic cells in mineralized and non-mineralized bone, while TRAP marks the response of osteoclasts in bone resorption in mineralized bone. These markers are used to evaluate the histological response to mineralization and maturation of the formed bone [57,58].

The enhanced presence of BMP 2 and BMP 4 around the biomaterials particles suggests that the biomaterials not only act as scaffolds for bone growth but also actively influence osteoblastic differentiation and matrix mineralization [25,30,59,60]. The more pronounced response observed in the G3/HSG can be attributed to its composition, which supports faster biomaterial resorption and enhances bone regeneration. This is evidenced by greater newly formed bone and less residual biomaterial in the G3/HSG.

In context, the histological findings of the two bioglasses implanted in this experimental protocol were compatible with osteoconductive properties, since, when in contact with body fluids, they present reactions that lead to the formation of a layer of carbonated hydroxyapatite. This formation occurs through a chemical bond between the material and the bone and is directly related to osteoconduction, guiding and supporting the growth of osteoprogenitor cells [61].

Finally, our morphometric analysis confirmed the qualitative and quantitative observations discussed above. The G3/HSG showed the highest percentage of new bone formation at 42 days, followed by the G2/SG group, both of which outperformed the control group. Moreover, the lower percentage of residual biomaterial observed in the G3/HSG suggests a more rapid and efficient resorption, which may be beneficial in clinical applications that require integration between the biomaterial and the host bone for long-term success. The process of reabsorption of biomaterial particles is a desired characteristic, in which this degradation process occurs concomitantly with the formation of new bone. This process is based on dissolution, which depends on the local pH and solubility, interfering with phagocytosis and the presence of leukocytes, as well as chemical mediators, which interfere with the local pH, reducing it. Other factors interfere with reabsorption, which can be faster with the increase in surface area, decrease in crystallinity, and changes in calcium sites [62]. Interestingly, Verdier et al. [63] reported the use of BioGlass 45S5 as an alternative to autografts in secondary alveolar bone grafting, achieving a success rate of over 80% after one year.

The effectiveness of SinGlass 45S5 and SinGlass High (F18), combined with their remodeling capacity and adequate mechanical support, makes them promising biomaterials for regenerating bone defects, thus meeting the clinical needs for autogenous graft substitutes. However, to validate the clinical application of these biomaterials in humans, it is advisable to conduct long-term follow-up studies and assess mechanical stability under diverse experimental conditions, as suggested in previous studies [44,64].

In summary, our results demonstrate that SinGlass 45S5 and SinGlass High (F18) have great potential for bone regeneration, exhibiting osteogenic and osteoconductive properties superior to those of the control group. The performance of the G3/HSG in bone regeneration suggests it could be a valuable alternative for bone repair and a viable substitute for autografts. Moreover, these biomaterials are known for their osteoconductive and osteoinductive properties, which facilitate the formation of a hydroxyapatite layer on the surface of bone defects. This layer provides an ideal foundation for bone growth and promotes adhesion between the biomaterial and the surrounding bone tissue [4,65].

Despite the promising results, this study has some limitations. The 42-day follow-up period may not have been sufficient to fully observe complete integration and resorption of the biomaterial, particularly for SinGlass 45S5. Moreover, the use of a single animal model (rats) limits the extrapolation of the results to humans, and further studies in pre-clinical models and with longer follow-up periods are needed to assess the long-term behavior of these biomaterials. Lastly, although qualitative and quantitative analyses showed promising results, complementary evaluation methods are recommended to better understand the mechanisms of action of these biomaterials and optimize their use in clinical applications.

Therefore, future studies with longer follow-up periods and more complex preclinical models are crucial to validate the clinical efficacy of these biomaterials. SinGlass High (F18) presents a viable alternative for bone regeneration, particularly in cases requiring autograft replacement, potentially reducing risks and promoting faster recovery.

## 5. Conclusions

SinGlass 454S5 and SinGlass High (F18) biomaterials showed high osteogenic and osteoconductive potential in rat calvarial bone defects. Both biomaterials promoted new bone tissue formation, with SinGlass High (F18) showing faster biomaterial resorption and a higher rate of bone maturation than SinGlass 45S5 and the control group. Their integration with the surrounding bone tissue was evident. Moreover, a favorable cellular response was induced, characterized by the presence of OCN, BMPs, and VEGF, suggesting a bioactive environment conducive to bone regeneration.

## Figures and Tables

**Table 1:** Percentages of new bone in the three experimental groups at 14 and 42 days. Control Group (G1/CG): bone defect filled with a blood clot. SinGlass Group (G2/SG): bone defect filled with approximately 0.010 g of SinGlass 45S5. High SinGlass Group (G3/HSG): bone defect filled with approximately 0.010 g of SinGlass High (F18).

Time Point		Group	
	G1/CG	G2/SG	G3/HSG
14 days	13.09 ± 0.80 ^A^	16.61 ± 2.65 ^B^	20.02 ± 2.84 ^BC^
42 days	17.54 ± 1.56 ^A^	31.66 ± 2.21 ^B^	36.28 ± 2.10 ^C^

Values are expressed as mean ± standard deviation. Group comparisons were conducted using ANOVA, followed by Tukey’s test for multiple comparisons, with statistical significance set at *p* < 0.05. Different letters indicate statistically significant differences among groups within the same period.

**Table 2:** Percentages of reaction tissue in the three experimental groups at 14 and 42 days. Control Group (G1/CG): bone defect filled with a blood clot. SinGlass Group (G2/SG): bone defect filled with approximately 0.010 g of SinGlass 45S5. High SinGlass Group (G3/HSG): bone defect filled with approximately 0.010 g of SinGlass High (F18).

Time Point		Group	
	G1/CG	G2/SG	G3/HSG
14 days	86.91 ± 0.80 ^A^	29.75 ± 21.18 ^B^	36.54 ± 16.00 ^BC^
42 days	82.46 ± 1.57 ^A^	39.83 ± 11.31 ^B^	28.05 ± 10.09 ^BC^

Values are expressed as mean ± standard deviation. Group comparisons were conducted using ANOVA, followed by Tukey’s test for multiple comparisons, with statistical significance set at *p* < 0.05. Different letters indicate statistically significant differences among groups within the same period.

**Table 3:** Percentage of residual biomaterial at 14 and 42 days in the two groups that received bioglass. SinGlass Group (G2/SG): bone defect filled with approximately 0.010 g of SinGlass 45S5. High SinGlass Group (G3/HSG): bone defect filled with approximately 0.010 g of SinGlass High (F18).

Time Point	Group	
	G2/SG	G3/HSG
14 days	53.64 ± 20.08 ^A^	43.48 ± 16.58 ^A^
42 days	82.46 ± 1.57 ^A^	39.83 ± 11.31 ^B^

Values are expressed as mean ± standard deviation. Group comparisons were conducted using ANOVA, with statistical significance set at *p* < 0.05. Different letters indicate statistically significant differences among groups within the same period.

## Data Availability

Data presented in this study are available on request from the corresponding author.

## Supplementary Materials

The following supporting information can be downloaded at: https://www.mdpi.com/article/10.3390/biom15010112/s1, Figure S1: Illustration demonstrating integration with osteoconductivity aspects of bioactive glass particles SinGlass (45S5) in (A) and SinGlass High (F18) in (B).
