# Supplementary material for: Biological Behavior of Bioactive Glasses SinGlass (45S5) and SinGlass High (F18) in the Repair of Critical Bone Defects
Source: Biomolecules. 2025 Jan 13;15(1):112. doi: 10.3390/biom15010112 (PMC11763790; doi:10.3390/biom15010112)
Supplement: Supplementary file 1 [file biomolecules-15-00112-s001.zip › biomolecules-3396801-supplementary.pdf]

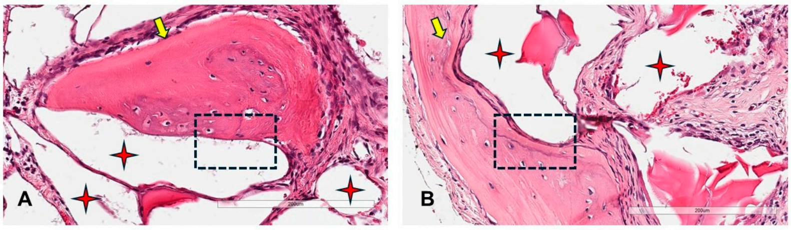

Figure S1: Illustration demonstrating integration with osteoconductivity aspects of bioactive glass particles SinGlass (45S5) in (A) and SinGlass High (F18) in (B). Biomaterial particles (red stars), new bone (yellow arrows), and integration area (black grid). Original magnification: Detail: 20×, scale bar = 200 μm.
